# Nutritional Contribution and Quality of Lunches Consumed During School Lunch Periods in Canadian Elementary Schools: A Plate Waste Analysis

**DOI:** 10.3390/nu18132065

**Published:** 2026-06-24

**Authors:** Natalia Alaniz-Salinas, Rachel Engler-Stringer, Hassan Vatanparast

**Affiliations:** 1 Department of Community Health & Epidemiology, University of Saskatchewan, Saskatoon, SK S7N 5E5, Canada; fex970@usask.ca; 2 College of Pharmacy and Nutrition, University of Saskatchewan, Saskatoon, SK S7N 5E5, Canada; vatan.h@usask.ca; 3 School of Public Health, University of Saskatchewan, Saskatoon, SK S7N 5E5, Canada

**Keywords:** school food programs, school meals, child nutrition, nutrition policy, population health, public health nutrition, food intake, dietary intake, plate waste

## Abstract

**Background/Objectives**: Foods and beverages consumed during school lunch periods contribute substantially to children’s dietary intake; however, Canadian evidence of their nutritional contribution and quality remains limited. This study assessed nutrient content, nutrient density, and contributions to dietary recommendations among Saskatchewan elementary students. **Methods**: A descriptive quantitative study was conducted among 379 students aged 5–13. Dietary intake during school lunch periods was assessed using a photography-assisted plate waste method. Nutrient content was estimated using standard nutrient databases, nutrient density was evaluated using the Nutrient-Rich Food (NRF) 9.3 Index, and contributions to dietary recommendations were examined. **Results**: Median lunch energy was 411.4 kcal (IQR: 296.7), and the mean NRF 9.3 score was 292.6 (SD: 130.7). Compared with home-packed and mixed lunches, school-provided lunches contained less energy, carbohydrate, fat, and sugar, while protein was similar across lunch types. Overall, lunches contributed <25% of daily requirements for key nutrients, including fibre, vitamin D, calcium, and potassium. Older students had lower proportional nutrient contributions relative to their higher nutritional requirements. Nutrient density differed by lunch provenance, but not by age or reported gender, with school-provided lunches achieving the highest NRF 9.3 scores. **Conclusions**: Lunches contributed modestly to daily nutrient requirements, particularly among older students. In this sample, school-provided lunches were associated with higher nutrient density than home-packed lunches, although their absolute contributions to several nutrients remained limited. These findings provide baseline evidence on lunches consumed during school lunch periods among Saskatchewan elementary students and may inform future evaluations of school food systems and policies.

## 1. Introduction

School-age children spend much of the weekday at school, where they consume food and beverages during breaks and at lunchtime. Adequate nutrition during childhood is essential for healthy growth, cognitive development, academic performance, and long-term health outcomes [1,2,3]. Internationally, school meal programs are increasingly recognized as important policy tools for improving diet quality and reducing nutrition inequities among children [4].

In Canada, about 26% of school-age children ate food prepared and served through a school lunch program during the 2023/2024 school year [5]. In 2024, the Government of Canada announced the development of a National School Food Program and subsequently released the National School Food Policy with a goal of providing more Canadian children with access to nutritious food through school-provided breakfasts, lunches and/or snacks [6]. Despite these recent policy developments, most students in Canada continue to rely on food brought from home, while participation in school food programs remains limited and varies across provinces and territories [7].

Evidence indicates that children’s diets in Canada are generally suboptimal, particularly during school hours, with frequent consumption of fast food, ultra-processed foods, and sugar-sweetened beverages, regardless of family socioeconomic status [1,8,9]. Previous Canadian studies have examined children’s dietary intake during school hours, school meals, and home-packed lunches, primarily using dietary recalls, food records, or questionnaire-based methods [1,10,11,12]. However, relatively little Canadian evidence has examined the nutritional contribution and quality of foods and beverages actually consumed during school lunch periods using objective measures of intake. In particular, few studies have used direct observation methods to evaluate lunchtime intake, compare lunch sources, and examine both nutrient contributions to dietary recommendations and overall nutrient density among elementary school students.

This study evaluated the nutritional contribution and quality of lunches consumed by public elementary school students in Saskatoon, Saskatchewan, Canada. The school food program offered in the participating school division was not universally available to all students and mostly focused on students being “in need”. The study aimed to (1) assess the energy and nutrient content of food and beverages consumed during school lunch periods; (2) evaluate their nutrient density using the Nutrient-Rich Food (NRF) 9.3 Index; (3) examine their contribution to age- and sex-specific dietary recommendations; and (4) compare these outcomes according to lunch provenance, age group, and reported gender.

## 2. Materials and Methods

### 2.1. Study Design

This study includes a descriptive quantitative analysis of lunches consumed during school lunch periods among elementary school students. The analysis used pre-intervention baseline data collected prior to the implementation of a universal school lunch program as part of a larger population health intervention research project.

### 2.2. Sample, Population, and Participants

This study forms part of the larger Good Food for Learning Population Health Intervention Research Project, which examines the implementation and impacts of a universal, curriculum-integrated school lunch program among elementary students living in low socio-economic neighbourhoods in Saskatoon, Canada. A protocol paper outlining the complete study has been published elsewhere [13].

The present analysis focuses exclusively on baseline data and does not evaluate intervention effects. Data were collected from four participating schools, purposively selected in collaboration with the local school division. All schools were located in lower socio-economic neighbourhoods and served ethnically diverse student populations, including higher proportions of Indigenous and newcomer children. Each school operated a small, self-managed lunch program, providing meals primarily to students identified as being in need, and supported through food donations and small grants [13].

All students from kindergarten to grade six enrolled in the selected schools were eligible to participate. The four participating schools enrolled approximately 620 students in kindergarten through grade 6 during the data collection period. Study records documented completed lunch observations, parental opt-outs, student absences, students who went home for lunch, and observations with missing or incomplete data. However, because attendance and participation varied across observation days and historical records were not available in a format that allowed reconstruction of participant flow, the total numbers of opt-outs, exclusions, and missing observations could not be reliably determined. Consequently, a complete participant flow from eligibility through inclusion could not be reconstructed.

Information describing the study was distributed to parents and caregivers before data collection. Guardians were given the opportunity to decline their child’s participation; students whose parents or caregivers did not opt out were considered eligible. On data collection days, researchers obtained verbal assent from students before taking measurements. The study received ethical approval from the University of Saskatchewan Behavioural Research Ethics Board and operational approval from the participating school division.

### 2.3. Sex- and Gender-Based Analysis

We recognize distinctions between sex and gender and their potential influence on nutritional requirements, intake, and eating behaviours. Dietary Reference Intakes (DRIs) are based on biological sex; however, the available data included only reported gender as provided by the schools. Consequently, reported gender was used as a proxy for sex when applying sex-specific DRI values and conducting subgroup analyses. Two participants had missing reported gender information and were retained in overall analyses but excluded from gender-stratified analyses and age–gender subgroup comparisons.

### 2.4. Data Collection, Instruments, and Variables

Data were collected in May and June 2021, with the aim of obtaining two lunch observations per student. Dietary intake was assessed using a digital photography–assisted plate waste method, following a standardized protocol. This method reduces reliance on participant recall and self-report and has been used previously to estimate food consumption and plate waste in school setting [14,15]. Students and families were not informed of observation days in advance to minimize behavioural changes [16].

On data collection days, all foods and beverages consumed during the lunch period were weighed and photographed immediately before consumption including items brought from home, items purchased externally, and items provided by the school lunch program. Students were asked not to share food or discard items during the lunch period. Field notes were recorded to document food provenance, packaging details, ingredients not visible in photographs, and any circumstances that could affect intake such as spills.

Pre- and post-consumption measurements were obtained using digital kitchen scales set to grams and tared prior to each measurement. Standardized photographs were taken of each food item while on the scale. Items were photographed individually, as grouped components within containers, or on school-provided plates. Photographs were captured at a 45-degree angle approximately 60 cm from the food, with the scale’s digital weight display and a participant identification label clearly visible within the frame. Identification labels were used to facilitate linkage of pre- and post-consumption images.

After lunch time, all remaining food, beverages, and packaging were weighed and photographed again to quantify plate waste. Intake was estimated as the difference between pre- and post-consumption weights. During data entry and cleaning, photographs and field notes were referenced for food identification and portion estimation and reviewed to support the interpretation of plate waste.

Each student was assigned a unique identifier to link dietary data with demographic information. Schools provided participants’ characteristics, including age, grade, and self-identified gender.

### 2.5. Data Management and Analysis

Researchers entered data from photographs and accompanying notes into a Microsoft Excel worksheet [17] to document food items, quantities consumed, and lunch provenance. Data were reviewed and cleaned by comparing pre- and post-consumption photographs with field notes. All entries were independently verified by a second researcher, and discrepancies were resolved through discussion. Only lunches with clearly identifiable food components were included in the analysis.

Nutrient analysis was conducted using Food Processor Nutrition and Fitness Software (version 11.6.522; ESHA Research, Salem, OR, USA) [18]. Food items were coded using the closest available match based on observed ingredients as determined through review of the research photographs and field notes. The Canadian Nutrient File (CNF) [19] was used as the primary source for food matching whenever suitable entries were available. USDA FoodData Central database [20] entries were used as a supplemental source, primarily for matching fruits, vegetables, and foods for which a more appropriate or updated match was available. Brand-specific products were not routinely coded; instead, nutritionally comparable generic food items were selected. For school-provided meals, recipes, ingredient lists, and preparation information provided by school nutrition staff were used to create food entries and estimate nutrient content. For home-prepared foods where recipe details were not available, coding was based on observed ingredients and standard recipes available within the software.

The analysis focused on energy intake and 18 nutrients of public health importance, including macronutrients (protein, fat, carbohydrates, dietary fibre, total sugar, and saturated fat), minerals (calcium, iron, magnesium, phosphorus, potassium, sodium, and zinc), and vitamins (vitamin A, folate, vitamin C, vitamin D, and vitamin E).

#### 2.5.1. Contribution to Dietary Reference Intakes

Lunches were compared to age- and sex-specific Dietary Reference Intakes [21] for selected nutrients, including fibre, vitamins, and minerals. Nutrient intakes were expressed as the percentage of the daily reference value provided by the lunch, using the Estimated Average Requirement (EAR) or Adequate Intake (AI), as appropriate, and the Chronic Disease Risk Reduction (CDRR) intake threshold for sodium. DRI reference values were obtained from Health Canada Dietary Reference Intakes Tables [21]. The reference values used for each nutrient and age/sex group are provided in Supplementary Table S1. These comparisons describe the relative contribution of lunch to daily nutrient requirements and do not represent overall dietary adequacy.

Energy and macronutrients were not included in the DRI contribution analysis because energy requirements vary according to individual characteristics such as age, body size, and activity level, whereas macronutrient recommendations are generally expressed relative to total energy intake rather than as fixed daily reference values.

#### 2.5.2. Nutrient Density Assessment

Nutrient density was assessed with the Nutrient-Rich Food Index 9.3 (NRF 9.3), a validated nutrient profiling model widely used to assess the nutritional quality of foods and overall dietary patterns [22,23,24,25]. The NRF 9.3 score was calculated as the sum of the percentage Daily Values (%DV) for nine nutrients to encourage minus the sum of the percentage Maximum Recommended Values (%MRV) for three nutrients to limit, standardized per 2000 kcal:

NRF 9.3 = NR9 − LIM3

where NR9 represents the sum of the percentage Daily Values for protein, dietary fibre, vitamin A, vitamin C, vitamin D, calcium, iron, magnesium, and potassium, and LIM3 represents the sum of the percentage Maximum Recommended Values for saturated fat, sodium, and total sugar.

For each nutrient, nutrient density was standardized to a 2000 kcal diet using the following equation:

%DV or %MRV = ((nutrient intake × 2000)/energy intake) ÷ reference value × 100

Consistent with conventional NRF methodology, individual nutrient contributions were truncated at 100% before summation to prevent disproportionately high values for any single nutrient from dominating the overall score [23]. Higher NRF 9.3 scores indicate greater nutrient density.

While the NRF 9.3 has a standardized structure, adaptations to specific nutrients have been described in previous applications to reflect regional dietary priorities and data availability [26]. In this study, minor modifications were implemented to better align the index with the Canadian nutritional context. Vitamin D was included in place of vitamin E among nutrients to encourage, given the high prevalence of inadequate vitamin D intake in Canada, particularly in northern populations with limited sunlight exposure [27]. This substitution aligns with Canadian dietary concerns and has been supported in previous applications of nutrient profiling models adapted to regional contexts [26,28]. In addition, total sugar was used in place of added sugar due to the absence of a specific quantitative recommendation for added sugars in Canadian dietary guidelines [29]. Previous work has shown that using total sugars instead of added sugars results in slightly lower, but still acceptable, correlations with overall diet quality indices [23]. Although this limits direct comparability with studies using the original NRF 9.3 formulation, the overall structure and interpretability of the index are preserved, as the model continues to balance nutrients to encourage and nutrients to limit within a standardized framework.

Daily values and maximum recommended values were based on Canadian guidelines for individuals aged four years and older, assuming a 2000 kcal diet [29]. These included dietary fibre (28 g), vitamin A (900 µg), vitamin C (90 mg), vitamin D (20 µg), calcium (1300 mg), iron (18 mg), potassium (3400 mg), magnesium (420 mg), total sugar (100 g), saturated fat (20 g), and sodium (2300 mg). Protein was benchmarked at 50 g per day, consistent with the Daily Value used in the original NRF methodology [23]. Although these reference values are not age-specific, they provide a standardized basis for comparison within the NRF framework, which is designed to evaluate nutrient density rather than individual adequacy.

NRF 9.3 scores were calculated in R 4.5.0 (R Core Team, 2025) [30] and linked to participant demographic characteristics and lunch provenance.

#### 2.5.3. Lunch Classification

Lunches were classified according to their source (provenance). Lunches provided entirely by the family or obtained from sources outside the school, such as items bought at a store, were classified as “home-packed”. Lunches provided by the school were classified as “school-provided”. Lunches containing items from both home and school sources were classified as “mixed” regardless of the relative contribution of each source.

For students with two observed lunch days, nutrient values from both observations were averaged at the participant level prior to analysis to avoid non-independence of repeated measures. Lunch provenance was subsequently assigned at the participant level based on the source(s) represented across the observation days contributing to the averaged intake. Participants whose lunch provenance differed across observation days (e.g., one home-packed lunch and one school-provided lunch) were classified as “mixed”. Consequently, the mixed category may include both lunches containing foods from multiple sources within a single observation day and participants whose lunch source varied across observation days. For students with only one observed lunch day, that observation was used for both nutrient calculations and lunch provenance classification.

### 2.6. Statistical Analysis

Energy intake, nutrient intakes, and NRF 9.3 scores were summarized descriptively using means (standard deviations [SD]) and medians (interquartile ranges [IQR]) for the total sample and means (SD) for subgroup analyses. Nutrient contributions relative to dietary reference values were summarized using means (SD).

Normality was assessed using Shapiro–Wilk tests and histogram inspection [31]. For normally distributed variables, independent-sample *t*-tests and one-way ANOVA were used; otherwise, Mann–Whitney U and Kruskal–Wallis tests were applied. When appropriate, post hoc pairwise comparisons were conducted using Dunn’s test with Bonferroni correction. All tests were two-tailed, with statistical significance set at *p* < 0.05. Given the descriptive and exploratory nature of the analyses, results should be interpreted cautiously, particularly for isolated statistically significant findings.

As a sensitivity analysis, a linear regression model including lunch provenance, age group, reported gender, and school fixed effects was fitted using NRF 9.3 score as the outcome to assess the potential influence of school-level clustering on the primary nutrient density findings.

## 3. Results

Plate waste data were collected from 379 students in kindergarten through grade 6, representing approximately 61% of kindergarten-to-grade-6 enrollment across the four participating schools. Measurements were obtained on two days for 68.1% of students and on one day for 31.9%. Participants ranged in age from 5 to 13 years, with a mean of 8.5 years (SD 1.9). Based on averaged lunch intake across observed school days, participants were classified according to the provenance of the lunches contributing to their averaged intake as school-provided (38%), home-packed (32.7%), or mixed (29.3%). Participant characteristics are presented in Table 1. The observation-level distribution of lunch provenance is presented in Supplementary Table S2.

### 3.1. Overall Caloric and Nutrient Intake

This study assessed 18 nutrients identified as being of public health importance. Table 2 presents the energy and nutrient content of lunches consumed during school lunch periods for the overall sample and stratified by reported gender. Table 3 provides a breakdown of nutrient content based on lunch provenance: school-provided, home-packed, or mixed. Absolute energy and nutrient intakes by age group are presented in Supplementary Table S3.

Lunches consumed during school lunch periods provided an average of 443.7 kcal (SD 217), with a median of 411.4 kcal (IQR 296.7). No statistically significant differences in total energy were observed by reported gender (*p* = 0.151). However, energy intake differed by lunch provenance, with school-provided lunches containing significantly less energy than both home-packed and mixed lunches (both *p* < 0.001).

Regarding macronutrients, school-provided lunches had significantly lower carbohydrate and fat content compared to the other lunch types (*p* < 0.05 for both). Protein content did not differ significantly by lunch provenance or reported gender, and contributed approximately 14% of total caloric intake across all groups. Sugar content was significantly lower in school-provided lunches than in home-packed and mixed lunches (both *p* < 0.001).

Dietary fibre intake averaged 4.1 g (SD 2.4), with males consuming slightly more fibre than females (*p* = 0.038). Saturated fat intake differed significantly by lunch provenance (*p* = 0.01), with school-provided lunches showing lower values overall.

Micronutrient profiles differed by lunch provenance. School-provided and mixed lunches generally provided higher amounts of calcium, vitamin A, vitamin D, and zinc than home-packed lunches. In contrast, folate, iron, and vitamin C were significantly higher in home-packed and mixed lunches than in school-provided lunches (all significant pairwise comparisons, *p* < 0.05).

### 3.2. Contribution to Daily Nutrient Recommendations

When expressed relative to Dietary Reference Intakes, lunches contributed a low proportion of daily nutrient requirements for several key nutrients (Table 4). Contributions were particularly low for fibre, vitamin D, potassium, and calcium, while higher relative contributions were observed for vitamin C and iron.

Nutrient contributions differed by lunch provenance. Home-packed and mixed lunches provided higher proportions of folate, iron, and vitamin C than school-provided lunches, whereas school-provided and mixed lunches contributed higher proportions of vitamin A and vitamin D than home-packed lunches (all significant pairwise comparisons, *p* < 0.05). Mixed lunches also provided the highest contributions for calcium, potassium, and zinc.

Differences were also observed by age group (Supplementary Table S4), with older children (9–13 years) generally showing lower proportional contributions relative to their higher nutritional requirements for several micronutrients, including calcium, folate, magnesium, phosphorus, and zinc, and vitamins A, C, and E (all *p* < 0.05). No statistically significant differences were observed by reported gender.

### 3.3. Nutrient Density of Lunches Consumed at School

The NRF 9.3 Index was used to assess the nutrient density of lunches consumed during school lunch periods (Table 5). The mean NRF 9.3 score was 292.9 (SD 130.7), with substantial variability across lunches. NRF 9.3 scores ranged from −32.7 to 675.6.

There were no statistically significant differences in nutrient density scores (NRF 9.3) between reported gender groups, between children aged 5–8 and those aged 9–13, or among age- reported gender subgroups. However, nutrient density scores differed significantly by lunch source: home-packed lunches had significantly lower NRF 9.3 scores than both school-provided and mixed lunches (both *p*-adjusted < 0.001). School-provided lunches also had higher scores than mixed lunches (*p*-adjusted < 0.001). Findings were similar in a sensitivity analysis excluding 73 participants whose lunch provenance differed across observation days (Supplementary Table S5). In this analysis, nutrient density remained significantly associated with lunch provenance (Kruskal–Wallis χ^2^ = 114.77, *df* = 2, *p* < 0.001), with school-provided lunches showing the highest NRF 9.3 scores, followed by mixed and home-packed lunches. In addition, sensitivity analyses adjusting for school fixed effects yielded similar results, with lunch provenance remaining significantly associated with NRF 9.3 scores (*p* < 0.001).

When examining the distribution across NRF 9.3 tertiles, no significant differences were observed by reported gender, age group, or age– reported gender subgroup. In contrast, lunch type differed significantly across tertiles (Supplementary Table S6; *p* < 0.001). Among home-packed lunches, 62.9% were in the lowest tertile, while among school-provided lunches, 58.3% were in the highest tertile.

## 4. Discussion

To our knowledge, this is the first Canadian school-based study to apply a digital photography-assisted plate waste method to assess foods and beverages consumed during school lunch periods. By providing more objective measures of intake than self-reported dietary assessment methods, this approach reduces reliance on recall and reporting bias [14,15] and contributes evidence to the limited Canadian literature on children’s dietary intake during the school day.

In our sample, 38% of participants were classified as consuming school-provided lunches across all observed school days, while an additional 29% were classified as mixed, reflecting consumption of foods from both school and home sources. Although not directly comparable because of differences in measurement and classification, this level of exposure to school-provided food appears higher than the approximately 26% of Canadian school-age children reported to have eaten food prepared and served through a school lunch program during the 2023/2024 school year [5]. This may reflect the socioeconomic context of the participating schools, which are located in lower-income and ethnically diverse neighbourhoods and where school food initiatives were available. However, because individual-level socioeconomic and demographic information was not collected, the present study cannot assess access, equity, or the factors influencing participation in school food programs.

Carbohydrates contributed approximately 52–57% of total lunch energy, and fat contributed approximately 34–36%, depending on lunch provenance. Both values fall within the Acceptable Macronutrient Distribution Range (AMDR) for children aged 4–18 years (45–65% of energy from carbohydrates and 25–35% from fat) [32], although fat intake in some lunch categories approached the upper end of the recommended range. Protein contributed approximately 14% of total lunch energy across all lunch types. This proportion falls within the AMDR of 10–30% of total energy intake for children and adolescents aged 4–18 years [32], suggesting that protein intake at lunch was generally balanced relative to energy intake.

Canadian studies suggest that children consume approximately one-third of their daily energy intake during school hours [12,33], indicating that school-time eating represents an important opportunity to support nutritional adequacy. However, overall, the foods and beverages consumed during school lunch periods provided relatively low proportions of recommended intakes for several key nutrients, particularly fibre, vitamin D, potassium, and calcium. Although most nutrients contributed less than one-quarter of daily requirements, sodium intake from lunch represented approximately 49–55% of the age-specific Chronic Disease Risk Reduction (CDRR) threshold, indicating that a substantial proportion of the recommended maximum sodium intake was consumed during a single meal. This finding suggests that sodium intake was relatively high compared with recommended maximum levels, despite the generally modest contribution of lunch to overall nutrient requirements, consistent with previous evidence that sodium intake among Canadian children frequently exceeds recommended levels [34].

Lunch provenance was strongly associated with both nutrient intake and overall diet quality. School-provided lunches contained less energy, carbohydrates, fat, and sugar, while demonstrating higher nutrient density overall. In contrast, home-packed lunches provided higher amounts of some nutrients, including folate and iron, but were generally less balanced nutritionally. Mixed lunches showed intermediate patterns across most nutrients. Similar findings have been reported in previous studies in which school meals were associated with higher overall diet quality despite lower energy intake [1,33].

Older students consumed greater amounts of energy and several nutrients than younger students (Supplementary Table S3). For example, the mean lunch energy intake increased from 420 kcal among children aged 5–8 years to 468 kcal among those aged 9–13 years, with similar increases observed for protein, fibre, potassium, magnesium, and zinc. However, these increases were modest relative to the substantially higher dietary reference values applicable to older children. For some nutrients, including calcium and vitamin D, absolute intakes were nearly identical across age groups despite higher requirements among older students. Consequently, lunches contributed a smaller proportion of daily nutrient requirements among older students, suggesting that age-related differences in nutritional requirements should be considered when planning and evaluating school meals.

Nutrient density, assessed using the NRF 9.3 Index, differed significantly by lunch provenance, but not by age or reported gender. School-provided lunches consistently achieved higher NRF 9.3 scores, while home-packed lunches were more frequently represented in the lowest tertile. Although school-provided lunches contained lower levels of some individual nutrients, including vitamin C, folate, and iron, they demonstrated higher nutrient density due to a more favourable balance between nutrients to encourage and nutrients to limit relative to energy content. Because the NRF 9.3 Index reflects nutrient density rather than absolute nutrient adequacy, these findings should be interpreted alongside the observed differences in nutrient content and contributions to dietary recommendations. The lower energy content and lower contributions of some nutrients suggest that opportunities may exist to further strengthen school-provided lunches through adequate portion sizes and the inclusion of nutrient-rich foods such as fruits, vegetables, whole grains, and iron-rich foods. Nevertheless, the results suggest that, in this sample, school-provided lunches were associated with higher nutrient density than home-packed lunches, consistent with previous research showing that school meals are often associated with higher overall dietary quality than packed lunches despite differences in energy and nutrient content [33].

This study was conducted in a context where school food programs in Canada remain limited and most students rely on home-packed lunches. With the recent introduction of the National School Food Program, these findings provide baseline descriptive evidence regarding foods and beverages consumed during school lunch periods and their nutritional contributions. The results may serve as a point of comparison for future evaluations of school food initiatives and changes in school food environments over time.

This study has some limitations. Data collection occurred during May–June 2021, when COVID-19 public health measures remained in place in Saskatchewan schools. During this period, food service operations were subject to infection-prevention protocols that limited food sharing and favoured individually packaged foods and simplified meal service models. These restrictions may have influenced both the types of foods available through school lunch programs and the foods brought from home, potentially affecting nutrient composition and limiting the comparability of findings with non-pandemic school environments. Consequently, the results should be interpreted within the context of pandemic-era school food practices and may not fully reflect contemporary school food systems.

Dietary intake was assessed on one or two school days per participant and therefore represents a snapshot of intake rather than habitual dietary behaviour. Although averaging repeated observations improved the stability of estimates for many students, day-to-day variation in food choices may not have been fully captured. In addition, only lunch intake was assessed; therefore, total daily dietary intake could not be evaluated.

Study records documented absences, parental opt-outs, students who went home for lunch, and observations with missing or incomplete data; however, attendance and participation varied across observation days, and a complete participant flow diagram could not be reconstructed. Consequently, some degree of selection or attendance bias may have occurred. Because only four schools participated, multilevel modelling was not feasible. Sensitivity analyses excluding participants with inconsistent lunch provenance across observation days and incorporating school fixed effects produced similar findings, although residual school-level clustering may still have influenced some results.

Although adaptations were made to the nutrients included in the NRF 9.3 Index to reflect the Canadian context and available dietary guidance, direct comparability with studies using the original formulation may be limited. In addition, the purposive selection of schools located in lower-income neighbourhoods and serving ethnically diverse student populations limits the generalizability of the findings. Lunch provenance, food availability, participation in school food initiatives, and dietary patterns may differ in schools serving populations with different socioeconomic and demographic characteristics. Consequently, these findings should be interpreted as reflecting the circumstances of schools serving socioeconomically disadvantaged communities rather than all Canadian elementary school settings.

Despite these limitations, the use of digital photography-assisted plate waste analysis is a key strength, allowing for a detailed assessment of foods and beverages consumed during school lunch periods while reducing reliance on self-reported dietary data.

## 5. Conclusions

This study examined foods and beverages consumed during school lunch periods by elementary school students attending four urban schools in Saskatchewan. In this sample, school-provided lunches demonstrated higher nutrient density than home-packed lunches, although the overall contribution of lunches to daily nutrient requirements was modest for several nutrients, particularly among older children. Fibre, vitamin D, potassium, and calcium were consistently low relative to recommended intakes, while sodium contributed a substantial proportion of recommended maximum levels during a single meal.

These descriptive findings identify nutritional gaps during school lunch periods and differences in nutrient density according to lunch provenance. Given the purposive selection of four schools and the cross-sectional nature of the study, the findings should not be generalized beyond similar school settings. Nevertheless, the results provide baseline information on lunchtime dietary intake and may provide context for future evaluations of school food systems and school-based nutrition initiatives.

## Figures and Tables

**Table 1:** Characteristics of study participants (*n* = 379).

Variable	Category	Total *n* (%)
Reported gender	Females	181 (47.8)
	Males	196 (51.7)
	Missing	2 (0.5)
		
Age (years)	Mean (SD)	8.5 (1.9)
	Range	5–13
	5–8 years	192 (50.7)
	9–13 years	187 (49.3)
		
Number of Lunch Observations	1	121 (31.9)
	2	258 (68.1)
		
Lunch Provenance	School-provided	144 (38)
	Home-packed	124 (32.7)
	Mixed	111 (29.3)

Note 1: Values are presented as frequencies (*n*) and percentages (%) for categorical variables and as mean (standard deviation [SD]) or median (interquartile range [IQR]), as appropriate, for continuous variables. Percentages may not sum to 100 due to rounding. Note 2: Reported gender information was obtained from school records and was used as a proxy for sex when applying sex-specific Dietary Reference Intake (DRI) values. Two participants with missing reported gender information were excluded from gender-stratified analyses but retained in overall analyses.

**Table 2:** Energy and nutrient content of lunches consumed during school lunch periods, overall and by reported gender.

Variable	Total		Female		Male		*p*-Value
	Mean (SD)	Median (IQR)	Mean (SD)	Median (IQR)	Mean (SD)	Median (IQR)	
Energy (kcal)	443.7 (217)	411.4 (296.7)	430.5 (221.6)	395.8 (275.6)	455.9 (212.5)	420.2 (308.7)	0.151
Protein (g)	15.1 (7.4)	14.7 (9.6)	14.7 (7.3)	13.9 (9.5)	15.5 (7.6)	15.3 (9.5)	0.219
Fat (g)	17.3 (10.2)	15.6 (13.5)	17.2 (10.6)	14.6 (12.2)	17.5 (9.8)	17 (14.1)	0.41
Carbohydrates (g)	58.2 (32.2)	52.6 (42)	55.7 (32.4)	49 (40.8)	60.6 (31.8)	56 (42.6)	0.088
Dietary fibre (g)	4.1 (2.4)	3.7 (3.2)	3.9 (2.4)	3.5 (3.1)	4.3 (2.3)	4 (3.3)	**0.038**
Sugar (g)	24.5 (16.5)	20.3 (20.6)	22.8 (15.2)	19.3 (19.7)	26 (17.5)	21.1 (20.2)	0.111
Saturated fat (g)	6.1 (3.8)	5.4 (4.6)	6 (4)	5.2 (4.5)	6.2 (3.7)	5.8 (4.7)	0.369
Calcium (mg)	204 (124.7)	192.4 (169.8)	195.6 (116.7)	189.5 (172.2)	211.8 (131.5)	196.4 (169.7)	0.369
Folate (mcg DFE)	76.7 (57.7)	58.1 (75.8)	75.2 (60.3)	54.6 (74.1)	78 (55.2)	59.8 (75.2)	0.294
Iron (mg)	2.5 (1.6)	2.3 (2.1)	2.5 (1.6)	2.1 (1.8)	2.6 (1.6)	2.4 (2.3)	0.173
Magnesium (mg)	57.6 (27.5)	52.9 (38)	54.6 (28)	45.5 (38.2)	60.4 (26.8)	56.9 (36.5)	**0.006**
Phosphorus (mg)	289.4 (140.5)	272.5 (186.5)	282.5 (142.3)	263.3 (184.3)	295.7 (138.9)	279.7 (179.1)	0.329
Potassium (mg)	522.1 (224.2)	495.8 (261.6)	500.7 (222)	482.3 (260.8)	541.9 (225)	516.7 (250)	0.055
Sodium (mg)	844.9 (484.9)	800 (620.8)	845.7 (490.3)	787.4 (648.8)	844.1 (481.1)	818.3 (608.2)	0.742
Vitamin A (mcg RAE)	131 (142.5)	105.1 (141.4)	127.6 (161.5)	94.6 (142.5)	134.1 (122.7)	109.1 (135.2)	0.313
Vitamin C (mg)	24.6 (25.3)	15.5 (29.8)	24.2 (25.6)	15.4 (30.6)	24.9 (25.1)	15.8 (27.9)	0.752
Vitamin D (mcg)	1.1 (1)	0.7 (1.4)	0.9 (0.9)	0.7 (1.3)	1.2 (1.1)	0.8 (1.4)	0.133
Vitamin E (mg)	1.6 (1.1)	1.4 (1.5)	1.6 (1.1)	1.3 (1.4)	1.6 (1)	1.5 (1.4)	0.283
Zinc (mg)	1.9 (1)	1.8 (1.3)	1.9 (1)	1.7 (1.3)	2 (1.)	1.9 (1.3)	0.128

Note: Values are presented as mean (standard deviation [SD]) and median (interquartile range [IQR]). *p*-values represent differences between reported gender groups (female and male) and were obtained using independent samples *t*-tests or Mann–Whitney U tests, as appropriate based on data distribution. Statistically significant *p*-values (*p* < 0.05) are shown in bold.

**Table 3:** Energy and nutrient content of lunches consumed during school lunch periods by lunch provenance.

Variable	School-Provided Mean (SD)	Home-Packed Mean (SD)	Mixed Mean (SD)	*p*-Value
Energy (kcal)	354.5 (159) ^a^	505.2 (219.4) ^b^	490.6 (241.3) ^b^	**<0.001**
Protein (g)	15.3 (7.3) ^a^	14.3 (7.5) ^a^	15.8 (7.5) ^a^	0.204
Fat (g)	15.5 (9.5) ^a^	18.8 (10.8) ^b^	18.1 (10) ^ab^	**0.013**
Carbohydrates (g)	39.4 (18.2) ^a^	71.5 (30.5) ^b^	67.8 (36.4) ^b^	**<0.001**
Dietary fibre (g)	4 (2.1) ^a^	3.9 (2.3) ^a^	4.4 (2.7) ^a^	0.496
Sugar (g)	14.5 (8.3) ^a^	30.1 (16.8) ^b^	31.1 (18) ^b^	**<0.001**
Saturated fat (g)	5.3 (3.3) ^a^	6.5 (4.3) ^ab^	6.6 (3.8) ^b^	**0.01**
Calcium (mg)	213.8 (131.6) ^b^	169.2 (105.1) ^a^	230.3 (127.9) ^b^	**<0.001**
Folate (mcg DFE)	50.7 (36.3) ^b^	98.4 (62.1) ^a^	86.2 (62.4) ^a^	**<0.001**
Iron (mg)	1.9 (1.1) ^b^	3.1 (1.5) ^a^	2.8 (1.8) ^a^	**<0.001**
Magnesium (mg)	57.1 (25.2) ^a^	53.9 (25.9) ^a^	62.3 (31.5) ^a^	0.123
Phosphorus (mg)	291.2 (132.9) ^a^	269.1 (144.9) ^a^	309.5 (143.2) ^a^	0.065
Potassium (mg)	510.7 (195) ^ab^	491.6 (237) ^a^	571 (238.4) ^b^	**0.012**
Sodium (mg)	820 (454.1) ^a^	826.9 (483.2) ^a^	897.2 (524.4) ^a^	0.633
Vitamin A (mcg RAE)	166.7 (124.2) ^b^	83.6 (172.8) ^a^	137.6 (110.1) ^b^	**<0.001**
Vitamin C (mg)	15.4 (14.2) ^b^	29.9 (29.6) ^a^	30.6 (28) ^a^	**<0.001**
Vitamin D (mcg)	1.5 (1.2) ^b^	0.5 (0.5) ^a^	1.1 (0.9) ^b^	**<0.001**
Vitamin E (mg)	1.6 (1) ^a^	1.6 (1) ^a^	1.6 (1.2) ^a^	0.704
Zinc (mg)	2.1 (1) ^b^	1.7 (1) ^a^	2 (1) ^b^	**0.003**

Note: Values are presented as mean (standard deviation [SD]). *p*-values were obtained using Kruskal–Wallis tests. Different superscript letters within a row indicate statistically significant pairwise differences between lunch source groups based on Dunn’s post-hoc tests with Bonferroni correction (*p* < 0.05). Statistically significant *p*-values (*p* < 0.05) are shown in bold.

**Table 4:** Contribution of lunches consumed during school lunch periods to daily nutrient recommendations (% of DRI) by lunch provenance.

Variable	School-Provided Mean (SD)	Home-Packed Mean (SD)	Mixed Mean (SD)	*p*-Value
Dietary fibre (%)	14.4 (7.4) ^a^	15 (9) ^a^	16.8 (10) ^a^	0.272
Calcium (%)	22.4 (14) ^ab^	18.7 (11.7) ^a^	25.6 (15) ^b^	**0.002**
Folate (%)	23.9 (17.8) ^b^	51.8 (34.1) ^a^	44.6 (31.6) ^a^	**<0.001**
Iron (%)	36.5 (21.8) ^b^	64.4 (31.9) ^a^	59.8 (36.2) ^a^	**<0.001**
Magnesium (%)	36.9 (18.2) ^a^	39.4 (21.1) ^a^	44.7 (24.8) ^a^	0.07
Phosphorus (%)	44.3 (28.9) ^a^	48 (30.4) ^ab^	55.4 (35.3) ^b^	0.052
Potassium (%)	21.4 (8) ^ab^	21 (10.2) ^a^	24.4 (9.9) ^b^	**0.005**
Sodium (% CDRR threshold)	48.8 (26.4) ^a^	50.8 (28.6) ^a^	55 (31.5) ^a^	0.468
Vitamin A (%)	46 (33.4) ^b^	25.8 (60.4) ^a^	42.5 (35.8) ^b^	**<0.001**
Vitamin C (%)	49.9 (45.6) ^b^	111 (122.2) ^a^	108.2 (101.9) ^a^	**<0.001**
Vitamin D (%)	15.2 (11.8) ^b^	4.6 (4.8) ^a^	11.3 (9.1) ^b^	**<0.001**
Vitamin E (%)	20.7 (12.9) ^a^	23.3 (15.6) ^a^	21.9 (15.4) ^a^	0.516
Zinc (%)	37.9 (19.2) ^ab^	34.2 (18.6) ^a^	40.9 (22.4) ^b^	**0.033**

Note: Values are presented as mean (standard deviation [SD]). Nutrient contributions are expressed as percentages of age- and sex-specific Dietary Reference Intakes (DRIs) based on the Estimated Average Requirement (EAR) or Adequate Intake (AI), as appropriate. Sodium is expressed as a percentage of the Chronic Disease Risk Reduction (CDRR) threshold rather than a DRI; higher percentages indicate greater contribution toward the recommended maximum sodium intake and should not be interpreted as a favourable nutritional contribution. *p*-values were obtained using Kruskal–Wallis tests. Different superscript letters within a row indicate statistically significant pairwise differences between lunch source groups based on Dunn’s post hoc tests with Bonferroni correction (*p* < 0.05). Statistically significant *p*-values (*p* < 0.05) are shown in bold.

**Table 5:** Nutrient density of lunches consumed during school lunch periods assessed using the NRF 9.3 index, overall and by sociodemographic characteristics and lunch provenance.

Category	Group	Mean (SD)	Median (IQR)	*p*-Value
Overall	–	292.9 (130.7)	282.3 (187.9)	
Reported Gender	Female	288.9 (131.2)	283.8 (181.8)	0.645
	Male	297.3 (130.9)	285.1 (190.9)	
Age Group	5–8 years	287.7 (130.3)	279.4 (195.1)	0.442
	9–13 years	298.2 (131.3)	287.8 (186.1)	
Age/Reported Gender Subgroup	Female 5–8 years	290.9 (130.2)	285.3 (192.8)	0.630
	Female 9–13 years	286.6 (133.1)	276.9 (164.2)	
	Male 5–8 years	285.8 (132.1)	272.4 (198.3)	
	Male 9–13 years	307.5 (129.7)	299.5 (201.4)	
Lunch Provenance	Home-packed	198.9 (107.9)	187.9 (129)	**<0.001**
	Mixed	294.3 (104.7)	287.2 (131.6)	
	School-provided	372.6 (112.5)	376 (183.5)	

Note: Values are presented as mean (standard deviation [SD]) and median (interquartile range [IQR]). *p*-values represent differences across categories and were obtained using independent samples *t*-tests, one-way analysis of variance (ANOVA), or Kruskal–Wallis tests, as appropriate based on data distribution. For lunch provenance, post hoc pairwise comparisons were conducted using Dunn’s test with Bonferroni correction. Statistically significant *p*-values (*p* < 0.05) are shown in bold.

## Data Availability

Dataset available on request from the authors due to ethical restrictions; participant consent did not include permission for public data sharing.

## Supplementary Materials

The following supporting information can be downloaded at https://www.mdpi.com/article/10.3390/nu18132065/s1. Table S1: Dietary reference values used to calculate nutrient contributions by age and sex-specific DRI category; Table S2: Observation-level distribution of lunch provenance across all lunch observations (*n* = 637); Table S3: Absolute energy and nutrient intake of lunches consumed during school lunch periods by age group; Table S4: Contribution of lunches consumed during school lunch periods to daily nutrient recommendations (% DRI) by age group and reported gender; Table S5: Sensitivity analysis of NRF 9.3 scores excluding participants whose lunch provenance differed across observation days; Table S6: Distribution of lunches consumed during school lunch periods across NRF 9.3 tertiles by lunch provenance.

## Abbreviations

The following abbreviations are used in this manuscript:

AMDR	Acceptable Macronutrient Distribution Range
CDRR	Chronic Disease Risk Reduction
CNF	Canadian Nutrient File
DRIs	Dietary Reference Intakes
DV	Daily Value
MRV	Maximum Recommended Value
NRF 9.3	Nutrient Rich Food 9.3
USDA	United States Department of Agriculture
