# Supplementary material for: Nutritional Contribution and Quality of Lunches Consumed During School Lunch Periods in Canadian Elementary Schools: A Plate Waste Analysis
Source: Nutrients. 2026 Jun 24;18(13):2065. doi: 10.3390/nu18132065 (PMC13363663; doi:10.3390/nu18132065)
Supplement: Supplementary file 1 [file nutrients-18-02065-s001.zip › nutrients-4360116-supplementary.pdf]

Supplementary Table S1. Dietary reference values used to calculate nutrient contributions by age and sex-specific DRI category.

Nutrient	Reference Type	4–8 years	Females 9–13 years	Males 9–13 years
Vitamin A (µg RAE)	EAR	275	420	445
Vitamin C (mg)	EAR	22	39	39
Potassium (mg)	AI	3800	4500	4500
Dietary fibre (g)	AI	25	26	31
Calcium (mg)	EAR	800	1100	1100
Vitamin D (µg)	EAR	10	10	10
Iron (mg)	EAR	4.1	5.7	5.9
Zinc (mg)	EAR	4	7	7
Folate DFE (µg)	EAR	160	250	250
Omega-3 (g)	AI	0.9	1.2	1
Omega-6 (g)	AI	10	12	10
Magnesium (mg)	EAR	110	200	200
Sodium (mg)	CDRR	1500	1800	1800

Note: EAR = Estimated Average Requirement; AI = Adequate Intake; CDRR = Chronic Disease Risk Reduction threshold. The 4–8 year DRI category was applied to participants aged 5–8 years.

Supplementary Table S2. Observation-level distribution of lunch provenance across all lunch observations (n = 637)

Lunch provenance	n (%)
School-provided	273 (42.9)
Home-packed	256 (40.2)
Mixed	108 (17)
Total	637 (100)

Supplementary Table S3. Absolute energy and nutrient intake of lunches consumed during school lunch periods by age group

Nutrient	5–8 years Mean (SD)	9–13 years Mean (SD)
Energy (kcal)	420.3 (203.7)	467.7 (227.9)

Protein (g)	14.1 (6.6)	16.1 (8)
Fat (g)	16.2 (9.6)	18.5 (10.7)
Carbohydrate (g)	55.9 (31.2)	60.7 (33)
Dietary fibre (g)	3.7 (2.1)	4.5 (2.6)
Total sugar (g)	24.7 (16.8)	24.2 (16.3)
Saturated fat (g)	5.9 (3.6)	6.2 (4.1)
Calcium (mg)	204.6 (116.2)	203.4 (133.1)
Folate DFE (µg)	74 (55)	79.4 (60.3)
Iron (mg)	2.39 (1.44)	2.71 (1.65)
Magnesium (mg)	53.9 (25.4)	61.4 (29.1)
Phosphorus (mg)	277.2 (127.5)	301.9 (152)
Potassium (mg)	490.9 (208.7)	554.1 (235.3)
Sodium (mg)	769.7 (414.3)	922.1 (538.3)
Vitamin A (µg RAE)	121.8 (154.6)	140.4 (128.7)
Vitamin C (mg)	23.8 (26)	25.3 (24.6)
Vitamin D (µg)	1.04 (0.92)	1.07 (1.12)
Vitamin E (mg)	1.43 (0.98)	1.78 (1.11)
Zinc (mg)	1.76 (0.87)	2.10 (1.08)

Note: Values are presented as mean (SD). Nutrient intakes represent foods and beverages consumed during school lunch periods. Corresponding contributions to age- and sex-specific Dietary Reference Intakes (DRIs) are presented in Supplementary Table S4.

Supplementary Table S4. Contribution of lunches consumed during school lunch periods to daily nutrient recommendations (% DRI) by age group and reported gender.

Variable	Age Group			Reported Gender		
	5-8 years Mean (SD)	9 to 13 years Mean (SD)	p-value	Female Mean (SD)	Male Mean (SD)	p-value
Dietary fibre (%)	14.8 (8.4)	15.8 (9.1)	0.298	15.3 (9.4)	15.3 (8.2)	0.534
Calcium (%)	25.6 (14.5)	18.5 (12.1)	**<0.001**	21.5 (13.2)	22.6 (14.4)	0.711
Folate (%)	46.2 (34.4)	31.8 (24.1)	**<0.001**	39.1 (32.2)	39.1 (29.2)	0.545
Iron (%)	58.2 (35.1)	46.6 (28.4)	**0.002**	52 (32.7)	53 (32.3)	0.497
Magnesium (%)	49 (23.1)	30.7 (14.6)	**<0.001**	39 (22.4)	40.9 (20.5)	0.141
Phosphorus (%)	68.4 (31.5)	28.6 (14.4)	**<0.001**	49.7 (31.7)	47.9 (31.6)	0.578
Potassium (%)	21.3 (9.1)	23 (9.7)	0.076	21.8 (9.7)	22.5 (9.2)	0.373
Sodium (%)	51.3 (27.6)	51.2 (29.9)	0.850	51.7 (28.7)	50.9 (28.9)	0.925
Vitamin A (%)	44.3 (56.2)	32.3 (29.5)	**0.014**	39.4 (55.5)	37.4 (33.6)	0.694

Vitamin C (%)	108.4 (118.4)	65 (63.1)	**0.010**	86.4 (100.1)	87.4 (95.4)	0.938
Vitamin D (%)	10.4 (9.2)	10.7 (11.2)	0.522	9.4 (8.9)	11.6 (11.2)	0.133
Vitamin E (%)	23.9 (16.3)	19.8 (12.3)	**0.049**	21.7 (14.8)	22.1 (14.4)	0.519
Zinc (%)	44 (21.8)	30.9 (15.8)	**<0.001**	37 (20.1)	38.1 (20.2)	0.509

Note: Values are presented as mean (SD). Nutrient contributions are expressed as percentages of Dietary Reference Intakes (DRIs). P-values represent differences between age groups (5–8 years vs. 9–13 years) and reported gender groups (female vs. male).

Supplementary Table S5. Sensitivity analysis of NRF 9.3 scores excluding participants whose lunch provenance differed across observation days.

**Lunch provenance**	**n**	**Mean (SD)**	**Median (IQR)**
Home-packed	124	198.9 (107.9)	187.9 (129)
Mixed	38	306.2 (89.9)	304.4 (93.9)
School-provided	144	372.6 (112.5)	376 (183.5)

Kruskal–Wallis $\chi^2 = 114.77$, $df = 2$, $p < 0.001$.

Supplementary Table S6. Distribution of lunches consumed during school lunch periods across NRF 9.3 tertiles by lunch provenance.

**Lunch Provenance**	**T1 (lowest) n (%)**	**T2 n (%)**	**T3 (highest) n (%)**	**p-value**
Home-packed	78 (62.9%)	36 (29%)	10 (8.1%)	
Mixed	31 (27.9%)	48 (43.2%)	32 (28.8%)	
School-provided	18 (12.5%)	42 (29.2%)	84 (58.3%)	**<0.001**

Note: Values are presented as n (%), with percentages calculated within lunch provenance. Tertiles are based on NRF 9.3 scores. P-value represents the chi-square test comparing the distribution of lunch types across tertiles.
